# Modeling Extracellular Potentials in Microelectrode Array Recordings

**DOI:** 10.1186/1471-2202-14-S1-P120

**Published:** 2013-07-08

**Authors:** Torbjørn B Ness, Espen Hagen, Moritz Negwer, Rembrandt Bakker, Dirk Schubert, Gaute T Einevoll

**Affiliations:** 1 Dept. of Mathematical Sciences and Technology, Norwegian University of Life Sciences, Ås, Norway; 2 Dept. of Cognitive Neuroscience, Donders Inst. for Brain, Cognition & Behaviour, Radboud University Medical Centre Nijmegen, The Netherlands; 3 Dept. of Neuroinformatics, Donders Inst. for Brain, Cognition & Behaviour, Radboud University Nijmegen, The Netherlands; 4 Jülich Research Institute, Germany

## 

Microelectrode Array (MEA) measurements from *in vitro *slices has become an important research tool in neuroscience, however the interpretation of such recordings is not always straightforward. We have developed a modeling framework for emulating *in vitro *MEA recordings that takes into account both the measurement physics of the MEA set-up, and the underlying neural activity of the slice, resulting in simulated data that closely resembles experimental recordings. Our modeling framework may aid interpretation of experimental data by reproducing the experimental procedure *in silico*, make experimentally testable predictions, and produce test-data for validating various analysis methods such as CSD estimates and spike-sorting algorithms.

Our simulations are separated into two domains; the first step is simulations of neuronal activity in populations of multi-compartment model neurons, and secondly solving the electrostatic forward problem in the extracellular space. For the neuronal simulations we employ LFPy [1], a Python module built upon NEURON's Python interface [2] to obtain the transmembrane currents in every compartment of the model neurons. Then the Finite Element Method (FEM) is used to solve the Poisson equation from electrostatics and calculate the extracellular potentials in the 3D volume including the electrode sites, and test various approximation schemes. Hence, the effects of the electrodes can be assessed together with the impact of inhomogeneities and anisotropies of the extracellular medium in recordings. The approach is in principle applicable to any multicompartment neuron model (from e.g. ModelDB [3]), any neuron number or any MEA electrode set-up.

We will present our modeling framework, together with an investigation of the electrode effects on the measured signals. Then we will go on to present two different applications. Firstly, we have produced spike-sorting test-data to benchmark automated spike-sorting algorithms [4] used on MEA recordings. This project is part of an international coordinated effort where such test-data will be collected and made available at http://spike.g-node.org, allowing exchange of synthetic and experimental test-data with known underlying activity, and systematic benchmarking and comparison of spike-sorting algorithms applied to such data [5]. Secondly we will present a project where we have been studying the LFP signature of single neurons receiving varying, sub-threshold sinusoidal current input measured by MEAs in an acute brain slice setting [6]. The model output is compared to corresponding experimental data, which includes the detailed reconstruction of the excited neuron.
